# Sacubitril/valsartan-induced liver injury: A case report and literature review

**DOI:** 10.1097/MD.0000000000034732

**Published:** 2023-08-11

**Authors:** Ting Zhang, Jin-lian Cai, Jie Yu

**Affiliations:** a Department II of Respiratory and Critical Care in Jiangxi Provincial People’s Hospital, The First Affiliated Hospital of Nanchang Medical College, Nanchang, Jiangxi, China.

**Keywords:** case report, liver injury, sacubitril/valsartan

## Abstract

Sacubitril/valsartan (Entresto) is the first drug approved for the treatment of symptomatic chronic heart failure with reduced ejection fraction in adult patients. There have been no reports of hepatotoxicity secondary to sacubitril/valsartan administration. Here, we report the first case of severe liver injury caused by sacubitril/valsartan.

**Patient concerns:**

A 90-year-old female patient taking sacubitril/valsartan was admitted due to chronic heart failure. Subsequently, the patient developed serious liver injury with increased hepatic transaminases.

**Diagnosis:**

Drug-induced liver injury, sacubitril/valsartan-related. No liver injury caused by other reasons was observed after thorough examination. After the withdrawal of sacubitril/valsartan, the liver function of the patient gradually returned to normal.

**Interventions:**

We chose general liver protection methods to improve her hepatic function, including magnesium isoglycyrrhizinate at 100 mg daily and polyene phosphatidylcholine capsules at 456 mg 3 times daily. We consulted with a hepatologist to discuss the best plan for her treatment. The last, we stopped sacubitril/valsartan.

**Outcomes:**

After the withdrawal of sacubitril/valsartan, the liver function of the patient gradually returned to normal.

**Lessons:**

Sacubitril/valsartan-induced liver injury is very rare. Clinicians should pay particular attention to the possibility of hepatotoxicity during sacubitril/valsartan treatment.

## 1. Introduction

Sacubitril/valsartan was first proposed as a replacement for an angiotensin-converting enzyme inhibitors for the treatment of chronic heart failure^[1,2]^ as well as for essential hypertension.^[3]^ It is a combination of sacubitril, a neprilysin inhibitor, with valsartan, an angiotensin receptor blocker. Its main adverse effects are angioedema, hypotension, renal insufficiency and hyperkalemia.^[4]^ No documentation mentions that it may cause liver injury, though it is prohibited for patients with severe liver function impairment, biliary cirrhosis and cholestasis. Herein, we report the first case of severe liver injury following sacubitril/valsartan therapy in our hospital (Jiangxi Provincial People Hospital, The First Affiliated Hospital of Nanchang Medical College) along with a literature review.

## 2. Case presentation

**Figure 1.**
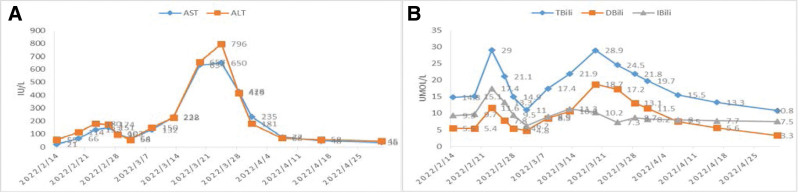
Includes 2 pictures (A and B), which mainly show the trends of some laboratory test results of this patient. For example, A shows the changes in alanine aminotransferase (ALT) and aspartate aminotransferase (AST) during the disease course, and B shows the changes in total bilirubin (TBili), direct bilirubin (DBili), and indirect bilirubin (IBili).

A 90-year-old female with a medical history of chronic obstructive pulmonary disease and chronic heart failure was admitted to our hospital due to chest tightness, severe shortness of breath and generalized edema on February 3, 2022. Her past medical history included tuberculous pleurisy and osteoporosis. She had no known diagnosis of chronic liver disease before she was hospitalized that day. She was not exposed to any new medication or herbal remedies in the 6 months prior to the onset of symptoms. She disavowed a history of drinking or smoking. There was no known history of liver disease in her family. According to the physical examination, her vital signs were normal. There were no bleeding points, spider angiomas or liver palms throughout her body. Her abdomen was flat and soft, with no tenderness or rebounding tenderness. Her liver and spleen were untouched. There was significant pitting edema over her eyelids, face and both legs. She began taking sacubitril/valsartan (100 mg twice per day) because of chronic heart failure on February 15. Liver chemistry assessed on February 14 revealed the following: total bilirubin (TBili) = 14.8 µmol/L (upper limit of normal (ULN) = 20.5 µmol/L), direct bilirubin (DBili) = 5.5 µmol/L (ULN = 6.84 µmol/L), indirect bilirubin (IBili) = 9.3 µmol/L (ULN = 13.66 µmol/L), aspartate aminotransferase (AST) = 55 U/L (ULN = 40 U/L), and alanine aminotransferase (ALT) = 21 U/L (ULN = 35 U/L). The laboratory results showed that all liver indices were in the normal ranges except AST. Four days after starting sacubitril/valsartan, repeat laboratory assessment revealed the following: TBili = 15.1 µmol/L, DBili = 5.4 µmol/L, IBili = 9.7 µmol/L, AST = 66 U/L, and ALT = 114 U/L. The levels of aminotransferases were slightly elevated. Therefore, we chose general liver protection methods to improve her hepatic function, including magnesium isoglycyrrhizinate at 100 mg daily and polyene phosphatidylcholine capsules at 456 mg 3 times daily. However, we found that her liver chemistry worsened (Fig. 1). At that time, we did not suspect liver injury secondary to sacubitril/valsartan therapy. For safety sake, we consulted with a hepatologist to discuss the best plan for her treatment. Viral hepatitis serologies were negative for hepatitis B virus (HBV) (HBsAb, HBeAg) and hepatitis C virus (HCVAb). The serologic markers of HBV HBsAg, HBcAg, and HBeAb were positive. Further laboratory testing demonstrated that the patient was negative for HBV DNA. Alpha-fetoprotein (1.8 ng/mL) was in the normal range (0–7 ng/mL). Abdominal ultrasound and computed tomography showed no abnormalities. This patient was not examined for hepatitis A IgM antibody or hepatitis E IgM antibody, as she was living in Nanchang, which was not an epidemic area of the abovementioned diseases. A liver biopsy was not performed on account of the poor general condition of this elderly patient.

On March 24, repeat laboratory assessment revealed the following: TBili = 24.5 µmol/L, DBili = 17.2 µmol/L, IBili = 7.3 µmol/L, AST = 796 U/L, and ALT = 650 U/L. Again, she had significantly elevated levels of aminotransferases. We considered that sacubitril/valsartan is prohibited for patients with severe liver function impairment, so the patient stopped oral sacubitril/valsartan. Over the first forty days after withdrawal, her AST and ALT levels decreased to normal little by little. The relationship between liver function and sacubitril/valsartan is shown in Figure 1. All evidence suggested that this patient liver injury was due to the sacubitril/valsartan. Although re-administration of sacubitril/valsartan to the patient would have confirmed its pathogenicity, sacubitril/valsartan was not readministered because of the severity of liver damage and her age.

## 3. Discussion and conclusions

Drug-induced liver injury (DILI) is an adverse reaction to drugs or other xenobiotics, which is likely to bring about severe clinical consequences, including acute liver failure, and may even be candidates for liver transplantation.^[5]^ Hepatic, neurological and cardiac toxicity cause 66% of the terminations during clinical testing.^[6]^ DILI has been divided into 2 varieties: intrinsic and idiosyncratic. The intrinsic type is dose-related and occurs shortly after exposure in most individuals exposed to the drug. The idiosyncratic kind is related to host factors and drug-host interactions. In a retrospective study to determine the incidence and causes of DILI in mainland China, the annual incidence in the general population was estimated to be 23.80 per 100,000 persons, higher than that reported in Western countries.^[7]^

In this case report, sacubitril/valsartan was the most likely cause of the patient liver function impairment. Her elevated transaminase levels came shortly after she began taking the drug. Viral hepatitis serologies were negative. Alpha-fetoprotein was in the normal range. The patient did not show signs, symptoms or imaging findings of alimentary diseases. Sacubitril/valsartan is a novel drug for the treatment of symptomatic chronic heart failure with reduced ejection fraction that combines the angiotensin receptor blocker valsartan and the neprilysin inhibitor prodrug sacubitril in a 1:1 ratio in a sodium supramolecular complex. Sacubitril is converted by esterases into LBQ657, which inhibits neprilysin, the enzyme responsible for the degradation of natriuretic peptides and many other vasoactive peptides.^[8]^ Thus, this combined angiotensin receptor antagonist and neprilysin inhibitor addresses 2 of the pathophysiologic mechanisms of heart failure (HF): activation of the renin-angiotensin-aldosterone system and decreased sensitivity to natriuretic peptides. It can decrease mortality and the risk of hospitalization in patients with HF.^[9,10]^ Liver dysfunction is prevalent in HF patients, so there has been growing interest in the relationship between the heart and the liver in HF.^[11]^ Sacubitril/valsartan improves measures of liver function compared with enalapril.^[12]^ One study determined the pharmacokinetics and safety of sacubitril/valsartan (LCZ696 [sacubitril/valsartan, Entresto, Novartis, C24H29N5O3.C24H29NO5.5/2H2O.3Na, molecular weight: 915.98]) in patients with mild and moderate hepatic impairment.^[13]^ Another study evaluated the effect of LCZ696 on portal-hypertensive rats. The researchers speculated that LCZ696 and valsartan reduced mean arterial pressure through peripheral vasodilation. LCZ696 significantly reduced portal pressure in portal vein ligation rats via hepatic endothelin-1 downregulation. LCZ696 could suppress the progression of diabetes-induced hepatic fibrosis, correlated with reduced oxidative stress, hepatic inflammation and nuclear factor kappa B, better than valsartan alone.^[14]^ Therefore, sacubitril/valsartan has a protective effect on the liver. There is no need for dose adjustment when administering Entresto to patients with slight hepatic injury, according to the literature on the rational use of medicines.

In this case, the patient was a 90-year-old woman. Owing to chronic heart failure, she had been taking sacubitril/valsartan (100 mg twice per day) on February 15. Later, we found that her liver chemistry worsened. Sacubitril/valsartan-induced liver injury was finally established based on the Naranjo adverse drug reaction probability scale^[15]^ and the Roussel Uclaf Causality Assessment Method (RUCAM) scale.^[16]^ The Naranjo adverse drug reaction probability scale involves 10 “yes,” “no,” or “unknown/inapplicable” questions.

Based on the total score, a probability of definite, probable, or doubtful is given. Our patient score was 5, which indicated “probable” adverse drug reactions. The RUCAM has been tested repeatedly for accuracy and has shown high sensitivity and specificity. Within RUCAM, points are awarded for 7 components, where the criteria for scoring the first 3 of the 7 components are determined by the R ratio. In this case, the R ratio was 2.42, which meant that the hepatic injury was mixed. The RUCAM score was calculated as 6, which translates as “probable.” We suspected DILI for the following reasons: There is a reasonable correlation between sacubitril/valsartan and liver function lesions; There was no evidence that sacubitril/valsartan tablets could cause liver function damage in the China National Knowledge Infrastructure, PubMed and other databases or in drug instructions; Patients with worse liver function were given a longer with administration time. The liver function of the patient gradually recovered to normal levels after the withdrawal of this drug; her past medical history included tuberculous pleurisy and osteoporosis; advanced age was her specific risk factor. She was not exposed to any new medication or herbal remedies in the 6 months prior to the onset of her symptoms. However, Epstein–Barr virus, cytomegalovirus, and further examinations for HAV and HEV were not tested after the clinician empirically established the high likelihood of DILI, which also made the RUCAM and Naranjo scores not higher. There are still some limitations to this study. The pathogenesis of sacubitril/valsartan-induced liver toxicity remains unclear. The pathogenesis of DILI is complex and involves drug metabolism, mitochondrial function damage, the immune response, genetics and the environment.^[5]^ The association between drug metabolism genotype and liver injury has not been fully explored owing to the lack of genetic testing. The mechanism of hepatotoxicity of sacubitril/valsartan is not known but appears to be immunological, involving genetic variants, oxidative damage, and direct hepatoxicity via mitochondrial dysfunction.

In conclusion, this is the first case report of DILI caused by sacubitril/valsartan in a real-world setting. Clinicians should pay particular attention to the possibility of DILI during sacubitril/valsartan treatment.

## Author contributions

**Conceptualization**: Ting Zhang, Jinlian Cai, Jie Yu.

**Data curation**: Jinlian Cai.

**Supervision**: Jie Yu.

**Validation**: Jie Yu.

**Writing – original draft:** Ting Zhang.

**Writing – review & editing:** Ting Zhang.
